# Identification and Validation of miRNAs Associated with the Resistance of Maize (*Zea mays L*.) to *Exserohilum turcicum*

**DOI:** 10.1371/journal.pone.0087251

**Published:** 2014-01-29

**Authors:** Fangli Wu, Jianhong Shu, Weibo Jin

**Affiliations:** Institute of Bioengineering, College of Life Sciences, Zhejiang Sci-Tech University, Hangzhou, China; Sun Yat-sen University, China

## Abstract

Northern leaf blight, caused by the fungus *Exserohilum turcicum* (Pass.), is a major disease of maize (*Zea mays* L.). MicroRNAs (miRNAs) have been recently reported as gene expression regulators related to several stress responses; however, evidence of the role of miRNAs in plant response to biotic stresses is limited. In this study, the miRNA expression patterns in maize in response to *E. turcicum* stress were investigated using a plant miRNA microarray platform. A total of 118 miRNAs were detected in mock- and *E. turcicum*-inoculated leaves. Among these miRNAs, miR530, miR811, miR829, and miR845 were identified as new miRNAs in maize through a homology-based approach. The secondary structures and putative targets of these miRNAs were also predicted. In addition, four miRNAs were differentially regulated in response to *E. turcicum*: miR811, miR829, miR845, and miR408. The functional annotation of the predicted targets indicated that these stress-responsive miRNAs regulate metabolic, morphologic, and physiologic adaptations in maize seedlings at the post-transcriptional level. Four targets were negatively correlated with their corresponding miRNAs (miR811, miR829, and miR408). Furthermore, we have demonstrated for the first time that miR811 and miR829 confers a high degree of resistance to *E. turcicum*, which can be used in maize breeding programs.

## Introduction

Maize is one of the most important staple food crops in the world. Maize diseases are a major limiting factor in its production. Among these diseases, Turcicum blight, caused by *Exserohilum turcicum(E. turcicum)*, is considered as a severe disease especially in regions with cool climates, with temperatures ranging from 20°C to 25°C, relative humidity from 90% to 100%, and low luminosity [1]. In China, yield losses have approached approximately 50% in the northern regions where crops suffered overwhelming *E. turcicum* infections [2].

Compared with traditional chemical treatments, many recent studies increasingly concentrated on host plant resistance, which is a more effective and economical way of controlling leaf blight diseases. Most of the qualitative genes such as *Ht1*, *Ht2*, *Ht3*, *HtM*, and *HtN* are dominant or partially dominant in treating maize diseases [2]–[4]. However, the efficiency of these genes is declining because of the emergence and rapid spread of resistant strains [2]. Therefore, it is of great importance to resort to identifying new resistance sources from artificial and natural inoculation, and to determining the resistance types and levels possessed by the available breeder’s materials and introduced germplasms.

Accumulating evidence supports that microRNAs (miRNAs) are hypersensitive to abiotic or biotic stress, as well as to diverse physiologic processes [5], [6]. MiRNAs are approximately 21-nucleotide noncoding RNAs that serve a crucial role in post-transcriptional gene regulation by degrading target mRNAs in plants. MiRNA-guided gene regulation in plants is involved in multiple developmental processes [7], including organ polarity [8], leaf growth [9], sex determination [10], and male or female sterility [11]. Previous research showed that several plant miRNAs respond to biotic stresses and their targets are stress-related genes, which suggest that miRNAs are crucial to the stress response of plants [12]–[13].

Stress tolerance has been directly linked with the levels of miR398, which is transcriptionally downregulated by oxidative stress. Unlike in wild-type *Arabidopsis* plants, decreased miR398 expression in transgenic *Arabidopsis* lines improves tolerance to oxidative stress [14]. In addition, miR395 is reportedly involved in the sulfate starvation response and miR399 is involved in the inorganic phosphate starvation response [15]–[16]. In rice, miR169g was confirmed as the only drought-induced member of the miR169 family [17]. Moreover, miR399 and miR398 are involved in bacterial infections [18]–[19]. Li et al. [20] found that the Arabidopsis miRNA effector protein, Argonaute1 (AGO1), is required for a number of pathogen-associated molecular pattern-triggered immune responses. Thus far, the miRNA expression profiles of maize under fungal stress conditions have rarely been reported. Efforts to identify fungus-responsive miRNAs and determine their expression patterns would improve our understanding of their functions in stress adaptation.

Microarray technology has been applied in the high-throughput detection of gene expression and it has proven useful in miRNA expression assays [21]. In the current study, the effects of 348 probes on maize leaves under *E. turcicum* stress conditions were analyzed using a miRNA microarray. Thirteen miRNAs were detected in maize for the first time. Among them, four miRNAs were further validated as novel maize miRNAs by retrieving their precursors and performing RNA gel blot analysis. In addition, three of these four were found to be involved in the *E. turcicum*-induced stress response. Finally, we verified that miR811 and miR829 confers a high degree of resistance to *E. turcicum.*

## Materials and Methods

### Plants, *E. turcicum* Inoculation, and RNA Extraction

Physiological race 1 of *E. turcicum* that was conserved in our laboratory was inoculated into the maize line OH43. The plants were inoculated at the four-leaf to six-leaf stage of growth in a greenhouse. The inoculations were performed in the morning by pipetting two to three drops of conidial suspension into each plant whorl. After inoculation, the plants were kept at 100% relative humidity to ensure spore germination. *E. turcicum* spores appeared in the leaves 9 days post inoculation (dpi). Pools of leaves were harvested from more than one plant at 0, 1, 3, 5, 7, and 9 dpi. The samples were used to analyze the temporal miRNA expression patterns.

Total RNAs were extracted from leaf tissues using a TRIzol reagent (Invitrogen, Carlsbad, CA, USA), followed by RNase-free DNase treatment (Takara, Dalian, China). The RNA concentrations were quantified by a NanoDrop ND-1000 spectrophotometer.

### MiRNA Microarray Assay

Total RNAs isolated from the 9-dpi leaves of *E. turcicum*- and mock-inoculated plant leaves were sent to CapitalBio Corporation (Beijing, China) for miRNA microarray analysis. The purification of miRNA samples, labeling, and hybridization analysis were executed following the manufacturer’s instructions. Briefly, 60 µg of total RNAs was used to extract small-sized RNA with a miRNA isolation kit (Ambion, Austin, TX, USA). Fluorescein-labeled miRNAs were hybridized to each non-coding RNA microarray slide, which contained probes that were complementary to 348 nonredundant plant miRNAs in the miRBase Registry [22]. Each probe was spotted in triplicate in each slide and every sample was assayed in duplicate. The data were extracted using LuxScan (CapitalBio), and the differential miRNAs were selected using SAM (Significance Analysis of Microarrays ver. 3.0).

### Computational Identification of miRNA Precursors

The miRNA sequences were mapped to maize genomic sequences obtained from the NCBI Network (http://www.ncbi.nih.nlm.org/) using BLASTN with a maximum of two mismatches. A fragment of ∼260-nucleotide genomic sequence flanking the small RNA at both the 5′ and the 3′ ends were used to predict the secondary structure of the miRNA precursor (stem loop formation) using RNAstructure (http://rna.urmc.rochester.edu/RNAstruc ture.html).

### Prediction of miRNA Target

In plants, miRNAs recognize their target mRNAs by perfect or near-perfect base pairing. Computational sequence similarity algorithms were developed to identify potential miRNA targets. To predict the plant miRNA targets, a web-based computing system, psRNATarget [23], was queried using mature miRNA sequences. The predicted target expressed sequence tags (ESTs) were analyzed against the NCBI nucleotide or protein databases for functional annotation.

### Validation of the Identified miRNA with RNA Gel Blot

The 100 µg amount of total RNA for each sample was resolved on a 15% polyacrylamide/1x TBE/8 M urea gel and subsequently transferred to a GeneScreen membrane (NIN). DNA oligonucleotides that are perfectly complementary to candidate miRNAs were end-labeled with [γ-^32^P]ATP by T4 polynucleotide kinase (New England Biolabs) to generate high specific probes. Hybridization and washing procedures were performed as described [5]. The membranes were briefly air dried and then exposed to a phosphor imager.

### Validation of Mature miRNA Expression Profile via Stem-loop Reverse Transcription (RT)-PCR

The expression profiles of four *E. turcicum*-responsive mature miRNAs were assayed by stem-loop Reverse Transcription (RT) quantitative PCR (qPCR). The stem-loop RT primers were designed following the methods described by Chen et al. [24] and Varkonyi-Gasic et al. [25]. 500 ng of total RNAs were used for initiating the RT of the mature miRNAs. The RT product was used as template for qPCR using a miRNA-specific forward primer and a universal reverse primer. The stem-loop RT reactions were performed using M-MLV Reverse Transcriptase (Takara, Japan) according to the supplier’s protocol. Primers were then added to perform PCR. U6, one of the uniformly expressed small RNAs, was used as the internal control for stem-loop RT-PCR. All the oligos used in this study were listed in supplemental table (Table S1).

SYBR Green PCR was performed as per the manufacturer’s instructions (Takara, Japan). Briefly, 2 µl of cDNA template was added to 12.5 µl of 2× SYBR Green PCR master mix (Takara), 1 µM concentration of each primer, and ddH_2_O to a final volume of 25 µl. The reactions were amplified for 10 s at 95°C, followed by 40 cycles of 95°C for 10 s and 60°C for 30 s. All reactions were performed in triplicate, and the controls (no template and no RT) were included for each gene. The threshold cycle (C_T_) values were automatically determined by the instrument, and the fold change of miR811 and miR845 was calculated using the following equation: 2^−ΔΔCt^, where ΔΔC_T_ = (C_T,target_−C_T,U6_)_Infection_−(C_T,target_−C_T,U6_)_Mock_ [26].

### Cloning and Sequencing of *E. turcicum*-responsive miRNAs

PCR primers were designed based on the retrieved pre-miRNA sequences using the Primer 5.0 program. An *Xba I* restriction site was added into the 5′ flanking region of the forward primer and a *Sac I* site was added to that of the reverse primer (Table S1). The PCR amplification was carried out using a programmable Thermal Cycler (BioRad, Washington DC, USA) using the following temperature program: initial denaturation at 96°C for 3 min; followed by 28 cycles of denaturation at 96°C for 30 s, annealing at 60°C for 30 s, and extension at 72°C for 30 min; and final extension at 72°C for 5 min and then maintained at 4°C. The amplification products were cloned into the pGEM-T Easy vector (Promega, Madison, USA), sequenced twice for every transformed colony to obtain credible pre-miRNA sequences.

### Expression Vector Construction and Disease-resistance Assessment of miRNA

The miRNA precursor cloned into pGEM-T was digested and introduced into the pBI121 vector through the *Xba I* and *Sac I* restriction sites. The resulting construct contained the miRNA precursor driven by the cauliflower mosaic virus 35S (*CaMV35S*) promoter and terminated by *nos* (Figure S1). The construct was introduced into *Agrobacterium tumefaciens* LBA4404 for maize transformation.

For the over expression of miRNA, Agrobacterium LBA4404 cells transformed with recombinant plasmid of pBI121-miRNA were cultured at 28°C in LB medium supplemented with kanamycin (50 µg/ml) and Rifampicin (20 µg/ml). When turbidity at 600 nm reached 1.0, the cells were collected by centrifugation at 5,000 rpm for 5 min and washed twice in resuspension buffer containing 10 mmol of MES (pH 5.6), 10 mmol of MgCl_2_, and 200 µmol of acetosyringone. The cell pellets were resuspended in resuspension buffer at an OD_600_ of 0.5 and then injected into the leaves of maize seedlings. The LBA4404 cells transformed with the clean pBI121 vector were also cultured and treated with the same process above as controls. After 24 hours, the grown *E. turcicum* agar discs (4 mm in diameter) were inoculated onto the injected leaves and the progression of *E. turcicum* infection was observed at 3 dpi. At the side of each *E. turcicum* agar disce, the leaf discs (4 mm diameter) were cut using a cork borer, frozen in liquid nitrogen and extracted the gDNA using plant DNA kit (Omega Bio-Tek Inc). Then the EtTubulin (*E. turcicum* Tubulin gene) was quantified using quantitative PCR, and the ZmTubulin (maize Tubulin gene) was used as the internal control for qPCR.

## Results

### miRNA Identified in Leaf Tissues by Microarray

MiRNA microarray analysis of the samples from the 9-dpi leaves of *E. turcicum*- inoculated and mock-inoculated plant was performed to investigate the potential involvement of miRNAs in *E. turcicum*-infected maize. The microarray was probed with pooled RNA isolated from several maize seedlings for each sample. A total of 118 of 348 miRNAs were detected in mock- and *E. turcicum* -inoculated leaves (Table S2). Of the 118, 105 miRNAs from 22 families had been previously identified in maize (Table 1) (miRBase Registry, Version 19) [22], whereas the remaining 13 miRNAs from 13 families were detected for the first time in maize leaves and considered as novel miRNA candidates (Table S3).

**Table 1:** Identification of known miRNA in maize leaves by miRNA microarray.

Family	Prob miRNA	Maize miRNA	Family	Prob miRNA	Maize miRNA
miR156	ath-miR156a,g	zma-miR156*a-l*	miR159	ath-miR159a,b,c	zma-miR159a-k
	zma-miR156k			osa-miR159a,c-f	
	ptc-miR156k			ptc-miR159e,f	
	osa-miR156k			sof-miR159c,e	
miR160	ath-miR160a	zma-miR160a-g	miR167	ath-miR167a,c,d	zma-miR167a-j
	osa-miR160e,f			osa-miR167d	
	ptc-miR160g,h			ptc-miR167f,h	
miR162	ath-miR162a	zma-miR162	miR390	ath-miR390a	zma-miR390a-b
miR164	ath-miR164a,c	zma-miR164a-h	miR393	ath-miR393a	zma-miR393a-c
	osa-miR164c-e			osa-miR393b	
miR166	ath-miR166a	zma-miR166a-n	miR171	ath-miR171a,b	zma-miR171a-n
	osa-miR166g,k,m			osa-miR171b,g,i,h	
	ptc-miR166n,p			ptc-miR171c,j	
	zma-miR166h			zma-miR171a,b,c	
miR168	ath-miR168a	zma-miR168a-b	miR169	ath-miR169d,h	zma-miR169a-r
	osa-miR168a,b			osa-miR169d-f,n,p,q	
	sof-miR168b			ptc-miR169ab,o,q,s,t,u,v,x	
miR319	ath-miR319a,c	zma-miR319a-d	miR399	mtr-miR399d	zma-miR399a-j
	osa-miR319a			osa-miR399h,i,j	
	ptc-miR319i			ath-miR399d,f	
miR394	ath-miR394a	zma-miR394a-b	miR408	ath-miR408	zma-miR408,b
miR396	osa-miR396d	zma-miR396a-h	miR397	ath-miR397a	zma-miR397a-b
	ath-miR396a,b			osa-miR397b	
miR172	ath-miR172a,c,e	zma-miR172a-e	miR398	osa-miR398b	zma-miR398a-b
	osa-miR172c			ath-miR398a	
	ptc-miR172g,i		miR528	osa-miR528	zma-miR528a-b
	sbi-miR172b		miR529	osa-miR529	zma-miR529
	zma-miR172a				

**Figure 1:**
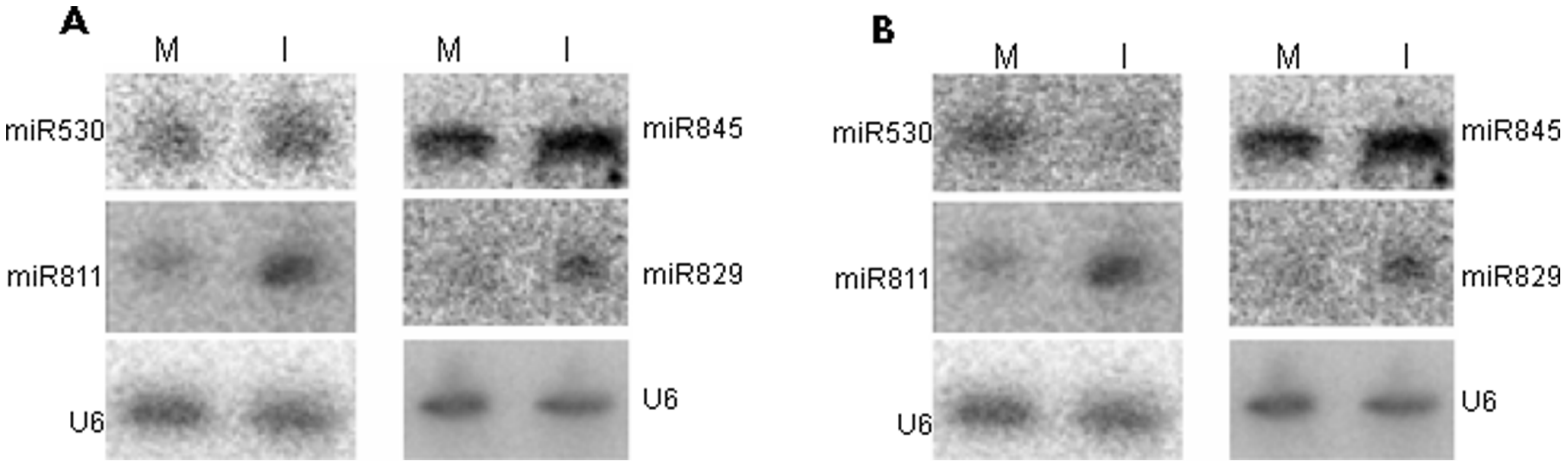
Detection of novel and differentially expressed miRNAs by northern blotting. A) Detection of novel miRNAs RNA; B) Detection of differentially expressed miRNAs. Northern blots of total RNA isolated from leaves of mock (M) and infected (I) leaves were probed with labeled oligonucleotides. The U6 RNA was used as internal control.

To confirm these 13 novel miRNA candidates, their precursor sequences were retrieved by mapping against the maize genome. Their secondary structures were predicted using the RNAstructure software. The results show that nine candidates could not fold into proper stem loop structures. The remaining four miRNA candidates, namely miR530, miR811, miR829, and miR845, were predicted to have good stem loop structures, as shown in the supplemental figure (Figure S2). The RNA gel blot was further analyzed to confirm these four miRNA candidates (Figure 1A). Therefore, these four miRNAs, miR530, miR811, miR829, and miR845 were considered as real novel miRNAs expressed in maize seedling leaves.

### *E. turcicum*-responsive miRNA Identified through Microarray Analysis

The differential expression between *E. turcicum* stressed leaves and mock leaves was analyzed for 109 miRNAs (105 reported and 4 novel miRNAs) that were identified in mock-inoculated and *E. turcicum*-inoculated samples. The result show more than twofold increases or decreases in expression at a *p* value <0.05. Four miRNAs on the microarray showed differential expression profiles in response to *E. turcicum* stress (Table 2). Moreover, three out of these four were novel miRNAs, namely miR811, miR829, and miR845. The remaining miRNA, miR408, has been previously identified in maize (miRBase Registry, Version 19) [22]. These results were also confirmed by the gel blot analysis (Figure 1B). Similar to the microarray data, gel blot findings show that miR811 and miR845 were upregulated in stressed leaves; miR408, which was expressed in mock-inoculated leaves, was not detected in *E. turcicum*-inoculated leaves; miR829, which was expressed in stressed leaves, was not detected in mock leaves (Figure 1B). These four miRNAs might contribute to the *E. turcicum* stress response of maize.

**Table 2:** *E.turcicum*-response miRNAs identified by microarray analysis.

Name	Expression change	Annotation
miR811	Up-regulation	Novel miRNA in maize
miR829	Expressed in stressed leaves, but not in mock leaves	Novel miRNA in maize
miR845	Up-regulation	Novel miRNA in maize
miR408	Expressed in mock leaves, but not in *E.turcicum*-inoculated leaves	Known miRNA in maize

### Expression Profiles of Stress-responsive miRNAs during Stress Treatments

**Figure 2:**
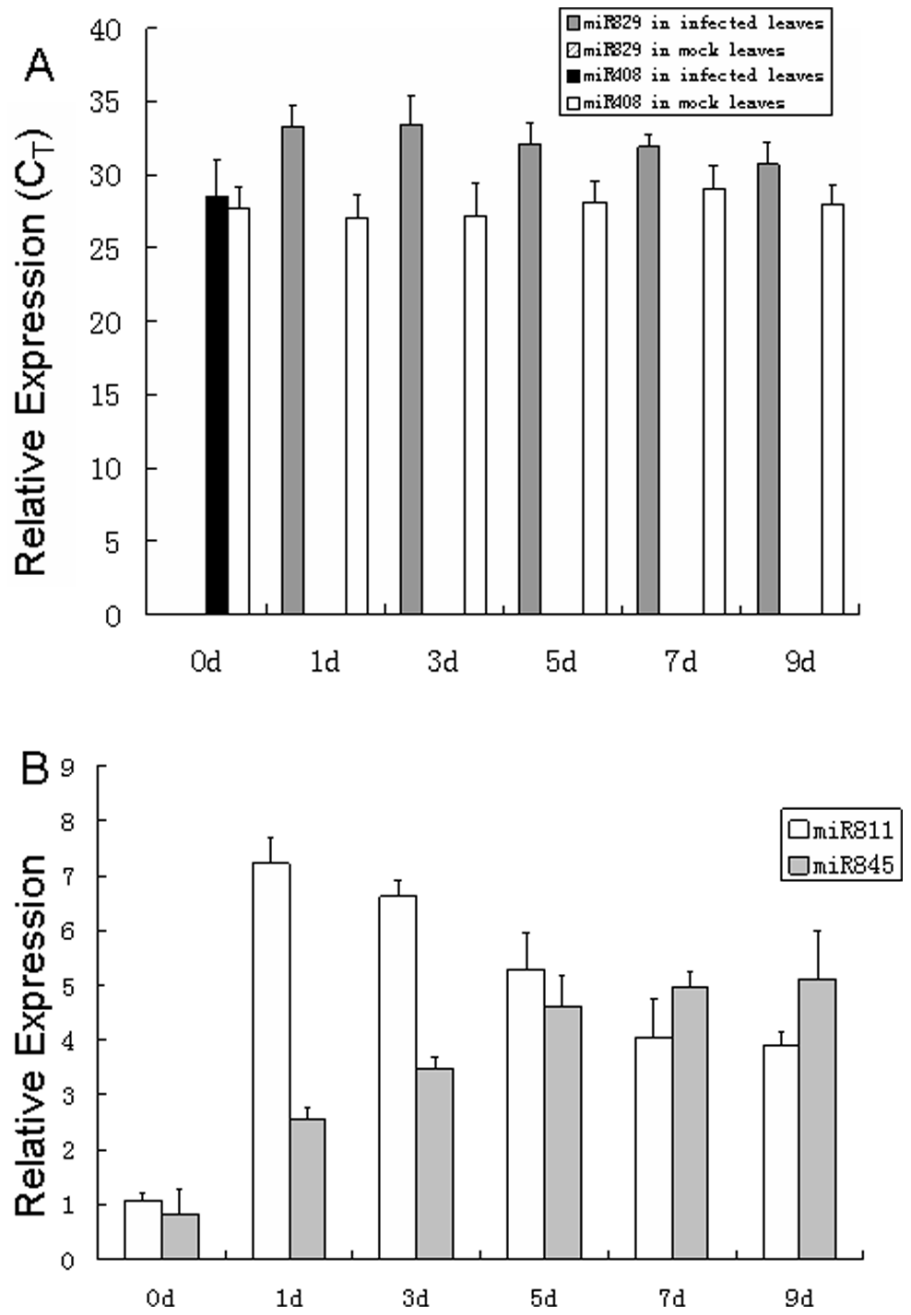
Quantitative analysis of miRNAs levels by stem-loop real-time RT-PCR. A) Expression profiles of miR829 and miR408 at 0, 1, 3, 5, 7 and 9 d; B) Expression of miR811 and miR845 at *E. turcicum*-infected leaves of 0, 1, 3, 5, 7 and 9 d using 2^−ΔΔCt^ method. U6 RNA was used as the internal control. Error bars indicate SD obtained from three biological repeats.

**Figure 3:**
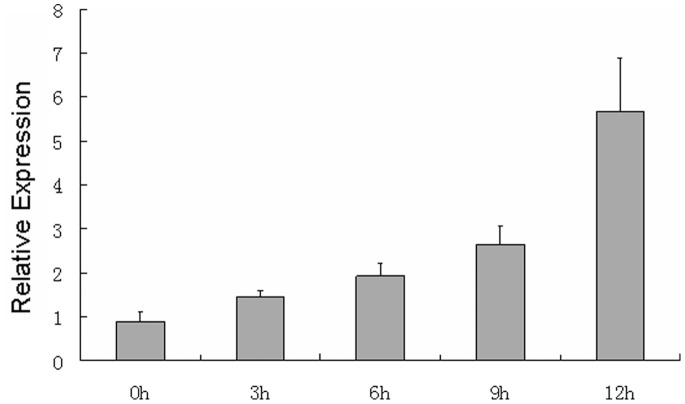
Quantitative analysis of miR811 levels by stem-loop real-time RT-PCR at 0, 3, 6, 9 and 12 h using 2^−ΔΔCt^ method. U6 RNA was used as the internal control**.** Error bars indicate SD obtained from three biological repeats.

Quantitative RT-PCR analysis was performed at six time points in healthy and infected leaves to determine the differential expression of miRNAs. The results were consistent with the microarray data. MiR408 expression was only detected in the control leaves, and miR829 was detected only in *E. turcicum-*inoculated leaves (Figure 2A). miR845 and miR811 were upregulated in the infected leaves at all time points (Figure 2B). Moreover, miR845 was slowly upregulated at all six time points in the infected leaves, but miR811 was upregulated to the maximization of approximately sevenfold in the 1 dpi leaves (Figure 2B). Thus, the expression of miR811 in the 1 dpi leaves was further analyzed at 0, 3, 6, 9, and 12 h after inoculation. As shown in Figure 3, miR811 expression was gradually upregulated within 12 h after inoculation.

### Potential Target Genes of Novel and *E. turcicum* Stress-responsive miRNAs

The sequences of four novel miRNAs (miR530, miR811, miR829, and miR845) and a differentially expressed known miRNA (miR408) were used to search against maize ESTs using psRNATarget to determine their potential regulatory targets [23]. A total of 14 target ESTs were predicted for the 5 miRNAs. We performed a target functional analysis based on the nucleic acid or protein databases of NCBI to understand better the biological function and classification of these miRNA targets, as well as their associated metabolic regulatory networks. The results are illustrated in Table 3. These targets could be classified into two categories: transcription factors and functional proteins.

**Table 3:** Prediction and function annotation of novel miRNAs.

miRNA_Acc.	Target Acc.	Target Description	Target Function
miR408	FL287021	similar to UniRef100_Q9LYQ2	Laccase-13 precursor
	TC523422	similar to AT1G22480	copper ion binding
miR530	FL221506	homologue to UniRef100_Q4W1F6	oxidoreductase activity
	CN844443	similar to UniRef100_Q0J1I2	Kinase activity
	BG268444	similar to UniRef100_Q0JJF5	RNA methyltransferase activity
	TC480219	similar to UniRef100_Q10LY8	DNA binding transcriptional factor
	TC529064	CNR 2	negative regulation of cell proliferation
	TC481499	similar to AT1G64210	protein phosphorylation
	TC513093	similar to AT3G59710	oxidoreductase activity
miR845	TC525988	similar to orf19.2669 of Candida albicans	DNA integration
miR811	TC473095	weakly similar to UniRef100_Q10FD6	Helix-loop-helix DNA-binding domain containing protein
miR829	TC508366	similar to UniRef100_Q0DA14	Os06g0684500 protein
	TC476722	similar to UniRef100_A7P5C4	adaptin region family protein
	TC500939	similar to UniRef100_A7NUQ8	Auxin-responsive factor TIR1-like protein

**Figure 4:**
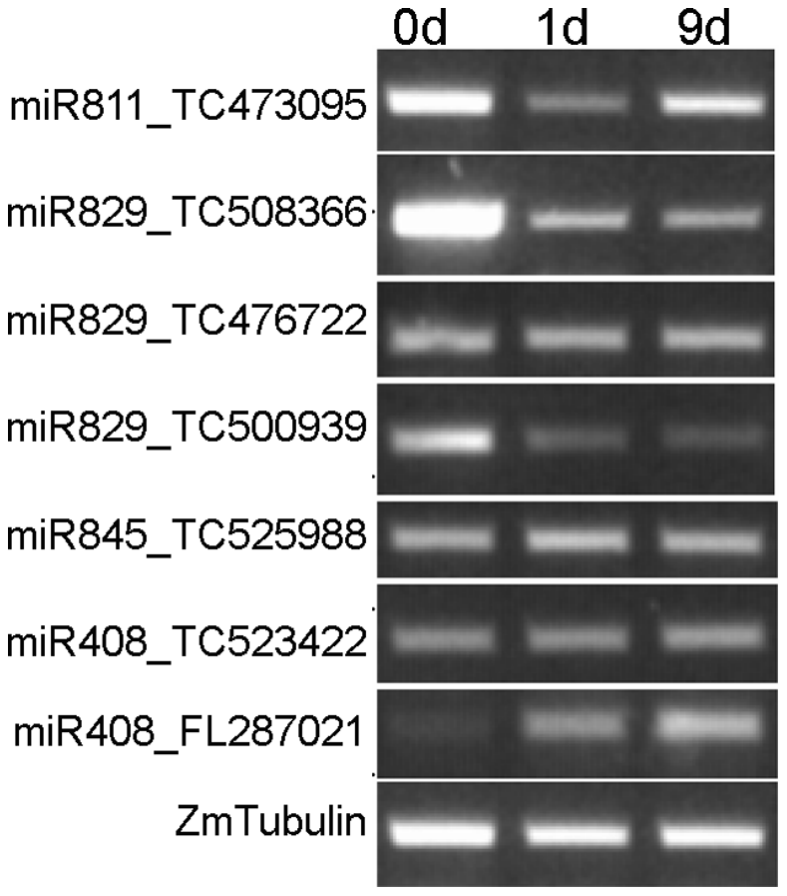
The expression of predicted targets were detectived using RT-PCR at 0 d, 1 d and 9 d. ZmTubulin was used as the inner control.

The transcription levels of seven mRNAs targeted by four differentially expressed miRNAs in *E. turcicum*-inoculated maize were investigated via RT-PCR at 0 d, 1 d, and 9 d after *E. turcicum* infection to understand their functions. As shown in Figure 4, the expression levels of the four targets (TC473095, TC508366, TC500939, and FL287021) were negatively correlated with three corresponding miRNAs (miR811, miR829, and miR408). By contrast, the remaining three targets (TC476222, TC525988, and TC523422) were uniformly expressed at different time points after *E. turcicum* infection. These results strongly suggest that miR811, miR829, and miR408 contribute to the *E. turcicum* stress response of maize.

### Disease-resistance Detection of *E. turcicum* -responsive miRNAs

To determine the disease resistance-related functions of miR811, miR829, and miR408, genomic DNA (gDNA) was extracted from maize leaves using the CTAB method. This gDNA was used as a template to amplify the precursors of miR811, miR829 and miR408 the amplification products were then sequenced for validation by the Invitrogen Co., Ltd. in China. Finally, 35S::miR811, 35S::miR829, and 35S::miR408 fusion constructs were produced by fusing these pre-miRNAs with the *CaMV35S* promoter (Figure S1). The fusion constructs were then introduced into *A. tumefaciens* LBA4404.

**Figure 5:**
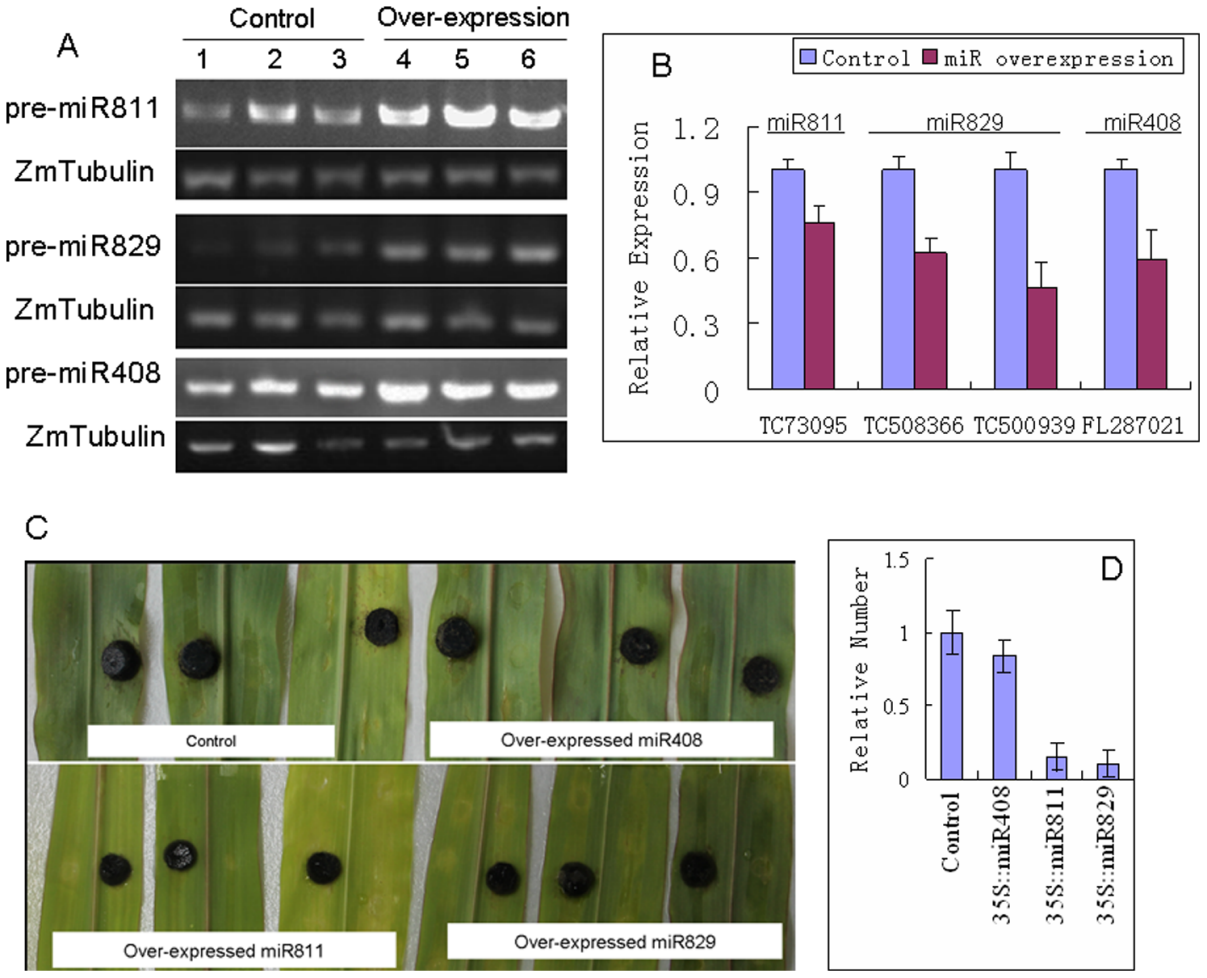
Overexpression and disease-resistance analysis of *E. turcicum*-responsive miRNAs. A) The expression levels of pre-miR811, pre-miR829, and pre-miR408 were detected in *Agrobacterium*-transformed leaves and control leaves; B) The expression of targets were analyzed in miRNA-overexpressing and control leaves; C) miRNA-overexpressing and control leaves were inoculated with *E. turcicum* agar discs (4 mm in diameter). Then, the maize leaves infected with *E. turcicum* were observed at 3 dpi. D) Analysis of *E. turcicum* amount in maize leaves. The EtTubulin was quantified using quantitative PCR, and the ZmTubulin was used as the internal control. Error bars indicate SD obtained from three biological repeats.

*A. tumefaciens* LBA4404 cells containing 35S::miR811, 35S::miR829, and 35S::miR408 were injected into the leaves of maize seedlings. LBA4404 cells with blank pBI121 vector were also cultured and injected into the maize leaves as controls. After 24 hours, the RT-PCR analysis showed that the three pre-miRNAs were overexpressed in the leaves transformed with the fusion vectors (Figure 5A). Meanwhile, the grown *E. turcicum* agar discs were separately inoculated onto miRNA-overexpressing and control leaves, and the infection of maize leaves by *E. turcicum* was observed at 3 dpi. The targets of these 3 miRNAs were down-regulated in miRNA-overexpressing leaves compared to control leaves (Figure 5B). Pathologic spots were noticeably reduced in the leaves that overexpressed miR811 and miR829 compared with those of miR408-overexpressing and mock-injected leaves (Figure 5C). To furtherly analyze the *E. turcicum* amounts, the EtTubulin was quantified using quantitative PCR. The result showed that the numbers of EtTubulins were obviously decreased in miR811- and miR829-overexpressing leaves compared to mock leaves, indicated that the growth of *E. turcicum* were inhibited on miR811- and miR829-overexpressing leaves compared to mock leaves (Figure 5D). These results strongly indicate that miR811 and miR829 enhance *E. turcicum* tolerance by regulating the expression of their target genes in maize leaves.

## Discussion

Microarray-based methods have been used to identify stress-responsive miRNAs in *Arabidopsis thaliana* [20], *Oryza sativa* [17], and tomato [27]. In the current study, we systematically identified miRNAs in mock-inoculated and *E. turcicum*-inoculated maize leaves. About one third of the probes (118 out of 348) in the microarray hybridized to miRNAs were present in maize. Moreover, the precursors of 4 of the 13 novel miRNA candidates that have been retrieved from maize genomic sequences with their putative stem loop structures were firstly detected in maize leaves. These 4 miRNAs, namely miR530, miR811, miR829, and miR845, proved to be novel miRNAs in maize via RNA gel blot analysis. The remaining nine miRNAs were considered “pseudo miRNA” because their precursors could not be retrieved from maize genomic sequences.

*E. turcicum* (Pass.) is an important pathogen in maize because it causes leaf blights, spots, and rots. However, despite the plethora of observed plant resistance mechanisms, the relationship between miRNAs and *E. turcicum* infection is still unclear. In this study, miR845 and miR811 were upregulated miRNAs in the treated leaves. Another miRNA, miR829, was detected in *E. turcicum*-stressed leaves, wherein miR408 was undetected. These four differentially expressed miRNAs are also involved in plant response to nutrient deficiency, drought, and other abiotic stress factors. Specifically, miR408 expression is upregulated in copper- and water-deficient plants [28]–[32], but decreased during iron starvation [29]. In contrast to induction under shock drought stress, miR829 is downregulated in hypoxic *Arabidopsis* [33], [34]. miR845 is upregulated under phosphorus deficiency in white lupin [35]. miR811 has not been implicated in the stress responses of plants, but its differential expression has been observed in the peripheral blood of lung cancer patients [36]. In the current study, the four gene targets (TC486069, TC476722, TC500939, and TC523422) of three miRNAs (miR811, miR829, and miR408) were negatively correlated with their corresponding miRNAs, as shown by the expression profiles of the seven target mRNAs at different time points. These results proved that the expression of miR811, miR829, and miR408 respond to *E. turcicum* infection in maize. In addition, the overexpression of miR811 and miR829 effectively improved *E. turcicum* tolerance by regulating target gene expression in maize leaves. Therefore, we have demonstrated for the first time that miR811 and miR829 confer a high degree of resistance to *E. turcicum*.

Previous research indicated that stress treatment triggers rapid changes in the transcript levels of plant miRNAs during the early stages of stress [17], [20], [37]. In *Arabidopsis*, the expression of miR156, miR167, miR168, and miR396 increased 2 h to 24 h after exposure to high-salinity treatment. The expression levels of miR167a increased after 2 h of drought stress, whereas the levels of most miRNA were higher after 6 h of inoculation and then decreased with prolonged cold stress treatment [20]. Moreover, even at 0.5 h after stress treatment, the transcript levels of some miRNAs were evidently altered [17], [37]. In our current study, the miRNA expression changed within 1 d after inoculation. This rapid change in miRNA expression quickly regulated the correlative functional genes to respond to the environmental stimuli, i.e., *E. turcicum*-induced stress. In addition, miR408 suppression and miR829 induction could be considered for further development as potential biomarkers for *E. turcicum* infection.

## Supporting Information

Figure S1 **Expression vector constructions of** ***E. turcicum*** **-responsive miRNAs.**
(PPT)

Figure S2 **The secondary structures of novel miRNAs.**
(PPT)

Table S1 All the primers used in this study.
(DOC)

Table S2 miRNA microarray data.
(CSV)

Table S3 Novel miRNAs candidates in maize via microarray.
(DOC)
