# Supplementary figures and images for: Identification and Validation of miRNAs Associated with the Resistance of Maize (Zea mays L.) to Exserohilum turcicum
Source: PLoS One. 2014 Jan 29;9(1):e87251. doi: 10.1371/journal.pone.0087251 (PMC3906166; doi:10.1371/journal.pone.0087251)

## Slide 1
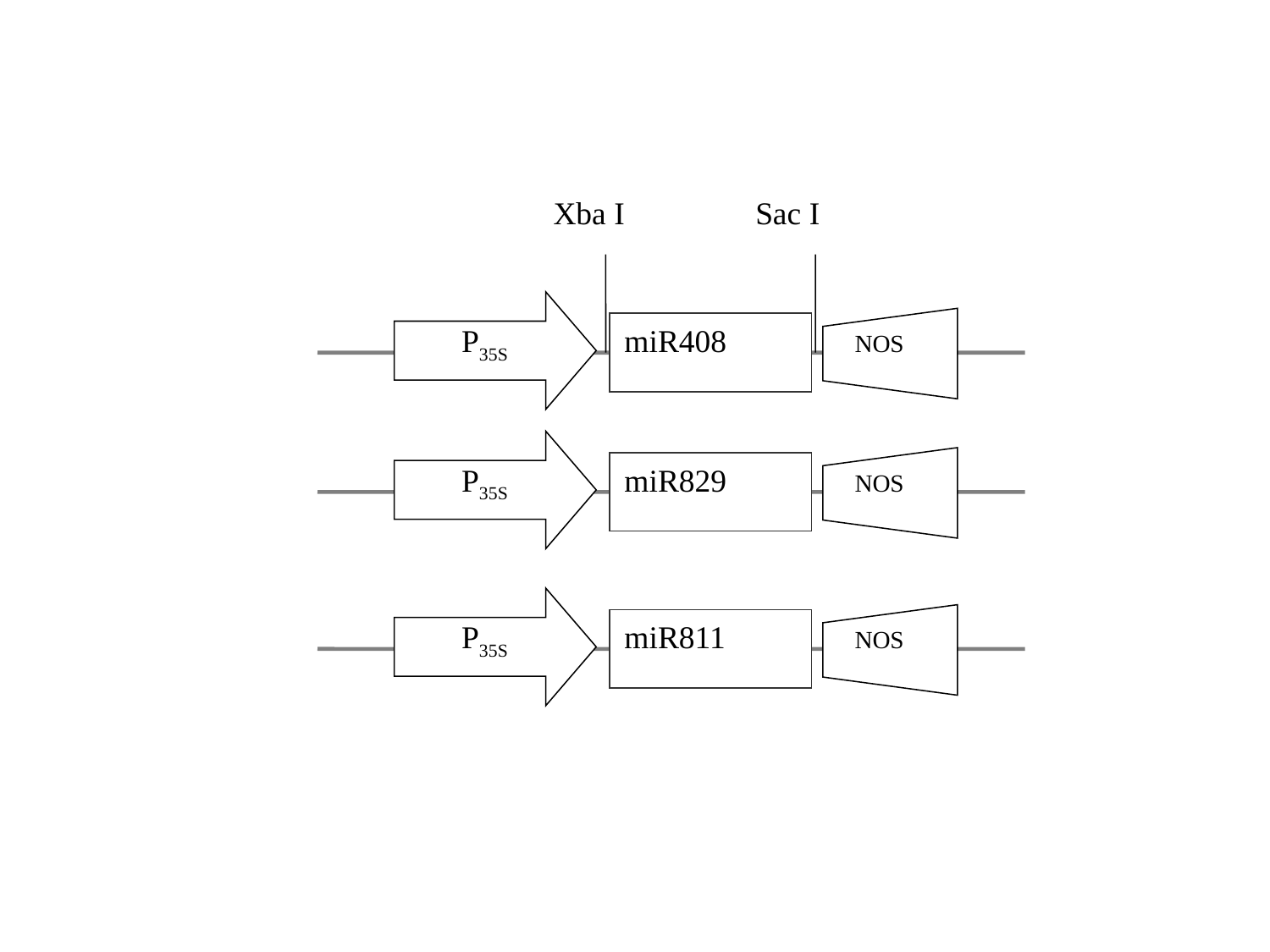

Xba I
Sac I
P35S
miR408
NOS
P35S
miR829
NOS
P35S
miR811
NOS

Supplement: Figure S1 — Expression vector constructions of E. turcicum -responsive miRNAs. (PPT) [file pone.0087251.s001.ppt]

## Slide 1
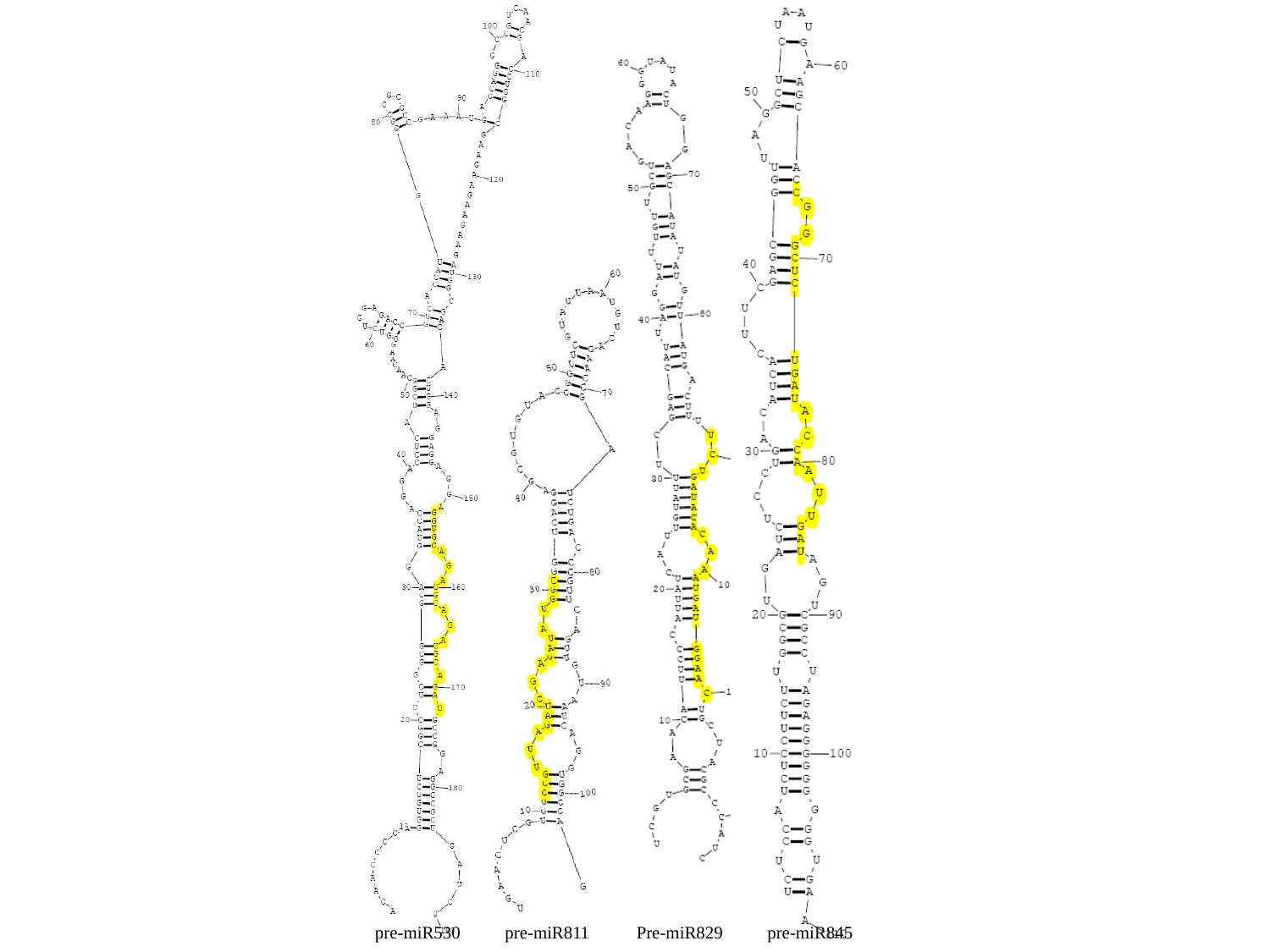

pre-miR530 pre-miR811
Pre-miR829 pre-miR845

Supplement: Figure S2 — The secondary structures of novel miRNAs. (PPT) [file pone.0087251.s002.ppt]
