# Supplementary material for: Identification and Validation of miRNAs Associated with the Resistance of Maize (Zea mays L.) to Exserohilum turcicum
Source: PLoS One. 2014 Jan 29;9(1):e87251. doi: 10.1371/journal.pone.0087251 (PMC3906166; doi:10.1371/journal.pone.0087251)
Supplement: Table S1 — All the primers used in this study. (DOC) [file pone.0087251.s003.doc]

Table S1 Primers used in this study.

| Target | Primer | sequence |
| --- | --- | --- |
| Quantitative RT-PCR miRNA | | |
| miR408 | RT | GTCGTATCCAGTGCAGGGTCCGAGGTATTCGCACTGGATACGACGCCAGG |
|  | Forward primer | GACTGCACTGCCTCTTC |
|  | Reverse primer | GTGCAGGGTCCGAGGT |
| miR811 | RT | GTCGTATCCAGTGCAGGGTCCGAGGTATTCGCACTGGATACGACTCCATA |
|  | Forward primer | GCGCCGTTAGATCGAGA |
|  | Reverse primer | GTGCAGGGTCCGAGGT |
| miR829 | RT | GTCGTATCCAGTGCAGGGTCCGAGGTATTCGCACTGGATACGACTTCCAT |
|  | Forward primer | GCTCTGATACACAAATG |
|  | Reverse primer | GTGCAGGGTCCGAGGT |
| miR845 | RT | GTCGTATCCAGTGCAGGGTCCGAGGTATTCGCACTGGATACGACATCAAT |
|  | Forward primer | CACGGGCTCTGATACCA |
|  | Reverse primer | GTGCAGGGTCCGAGGT |
| U6 | RT | GTCGTATCCAGTGCAGGGTCCGAGGTATTCGCACTGGATACGACAAAATATGGAAC |
|  | Forward primer | TGCGGGTGCTCGCTTCGGCAGC |
|  | Reverse primer | GGGCAGCCAAGGATGACT |
| Vector construction | | |
| 35S::miR408 | Forward primer | GGCA***TCTAGA***CAGGGACGAGGCAGAGCAT |
|  | Reverse primer | ATGG***GAGCTC***GAGCCAGGGAAGAGGCAGTG |
| 35S::miR811 | Forward primer | ACTG***TCTAGA***TGAACTCGTGGCCGTTAGA |
|  | Reverse primer | CGCC***GAGCTC***CTGGCCACCTGATTACAACT |
| 35S::miR829 | Forward primer | GGCG***TCTAGA***TCGTGCGAACATTCCCAT |
|  | Reverse primer | GCGG***GAGCTC***GATGGGCGTAGCAGTTCCA |
| Quantitative RT-PCR | | |
| C508366 | Forward primer | ATCTGTGAGCGAGGTAGGGA |
|  | Reverse primer | GGAGCTGGTGTTGATGACG |
| FL287021 | Forward primer | TGACATCGCCGTCCTCGTC |
|  | Reverse primer | TACCTGCCCGTTCACCGT |
| TC523422 | Forward primer | CGACGACGACGGACGAGT |
|  | Reverse primer | TCGGGGACGCAAGCAGAG |
| TC525988 | Forward primer | ATTTCAGTTTCGCCCTATTC |
|  | Reverse primer | CATCACTTCGAGCGGTTAGGC |
| TC473095 | Forward primer | GGATGAAATGCGAGATGAAA |
|  | Reverse primer | ACGTTGAGGCTACTGACTGC |
| TC476722 | Forward primer | AAACCCTCCCAAACAAGCTGT |
|  | Reverse primer | TTCAAATGGCCCACGATGC |
| TC500939 | Forward primer | ACTTGTGCTTTGTGCCATCT |
|  | Reverse primer | ATAGCGGAGTGGTCGAGTTT |
| ZmTubulin | Forward primer | TGTCGTCCAACCTTACAACTCACT |
|  | Reverse primer | TCTCCAGGGTCCTCCATTCC |
| EtTubulin | Forward primer | GGGAACTCCTCACGGATGTTG |
|  | Reverse primer | TAACAACTGGGCAAAGGGTCA |
