# Supplementary material for: Identification and Validation of miRNAs Associated with the Resistance of Maize (Zea mays L.) to Exserohilum turcicum
Source: PLoS One. 2014 Jan 29;9(1):e87251. doi: 10.1371/journal.pone.0087251 (PMC3906166; doi:10.1371/journal.pone.0087251)
Supplement: Table S3 — Novel miRNAs candidates in maize via microarray. (DOC) [file pone.0087251.s005.doc]

Table S3 Novel miRNAs candidates in maize via microarray

| Family | Prob miRNA | Family | Prob miRNA |
| --- | --- | --- | --- |
| miR391 | ath-miR391 | miR535 | osa-miR535 |
| miR414 | osa-miR414 | miR810 | osa-miR810 |
| miR416 | osa-miR416 | miR811 | osa-miR811a |
| miR439 | osa-miR439a | miR829 | ath-miR829.1 |
| miR476 | ptc-miR476b | miR845 | ath-miR845a |
| miR530 | osa-miR530 | miR854 | ath-miR854a |
| miR533 | ppt-miR533a |  |  |
